# Placental Element Content Assessed via Synchrotron-Based X-ray Fluorescence Microscopy Identifies Low Molybdenum Concentrations in Foetal Growth Restriction, Postdate Delivery and Stillbirth

**DOI:** 10.3390/nu16152549

**Published:** 2024-08-03

**Authors:** Vladimira Foteva, Kaushik Maiti, Joshua J. Fisher, Yixue Qiao, David J. Paterson, Michael W. M. Jones, Roger Smith

**Affiliations:** 1 Mothers and Babies Research Centre, Hunter Medical Research Institute, Newcastle 2305, Australia; kaushikmaiti@yahoo.com (K.M.); joshua.fisher@newcastle.edu.au (J.J.F.); roger.smith@newcastle.edu.au (R.S.); 2 School of Medicine and Public Health, University of Newcastle, Newcastle 2308, Australia; 3 Wisdom Lake Academy of Pharmacy, Xi’an Jiao Tong Liverpool University, Suzhou 215123, China; Yixue.Qiao@xjtlu.edu.cn; 4 Australian Synchrotron, Australian Nuclear Science and Technology Organisation, Clayton 3168, Australia; davidp@ansto.gov.au; 5 School of Chemistry and Physics, Queensland University of Technology, Brisbane 4000, Australia; mw.jones@qut.edu.au; 6 Central Analytical Research Facility, Queensland University of Technology, Brisbane 4000, Australia

**Keywords:** X-ray fluorescence microscopy, synchrotron, postdate placental tissue, foetal growth restriction, stillbirth, molybdenum

## Abstract

Placental health and foetal development are dependent upon element homeostasis. Analytical techniques such as mass spectroscopy can provide quantitative data on element concentrations in placental tissue but do not show spatial distribution or co-localisation of elements that may affect placental function. The present study used synchrotron-based X-ray fluorescence microscopy to elucidate element content and distribution in healthy and pathological placental tissue. The X-ray fluorescence microscopy (XFM) beamline at the Australian Synchrotron was used to image trace metal content of 19 placental sections from healthy term (*n* = 5, 37–39 weeks), foetal growth-restricted (*n* = 3, <32 weeks, birth weight <3rd centile), postdate (*n* = 7, >41 completed weeks), and stillbirth-complicated pregnancies (*n* = 4, 37–40 weeks). Samples were cryo-sectioned and freeze-dried. The concentration and distribution of fourteen elements were detected in all samples: arsenic, bromine, calcium, chlorine, copper, iron, molybdenum, phosphorous, potassium, rubidium, selenium, strontium, sulphur, and zinc. The elements zinc, calcium, phosphorous, and strontium were significantly increased in stillbirth placental tissue in comparison to healthy-term controls. Strontium, zinc, and calcium were found to co-localise in stillbirth tissue samples, and calcium and strontium concentrations were correlated in all placental groups. Molybdenum was significantly decreased in stillbirth, foetal growth-restricted, and postdate placental tissue in comparison to healthy-term samples (*p* < 0.0001). Synchrotron-based XFM reveals elemental distribution within biological samples such as the placenta, allowing for the co-localisation of metal deposits that may have a pathological role. Our pilot study further indicates low concentrations of placental molybdenum in pregnancies complicated by foetal growth restriction, postdate delivery, and stillbirth.

## 1. Introduction

Foetal development is reliant on the function of the placenta as a nutrient transporter, endocrine gland, and selective barrier against xenobiotic threats in the maternal circulation [1]. Abnormal placental function is associated with foetal growth restriction, preterm birth, and stillbirth [2,3,4,5,6]. As a component of the fetoplacental unit, the placenta is synthesised from nutrients provided by the mother that are composed of the elements within her diet and inspired air.

Various elements are required for the biosynthesis of the normal foetus and placenta, contributing to physiological processes within the cell, including gene transcription and expression and the development of organ systems and foetal structures [7]. Maternal dietary deficiency of micronutrient elements such as zinc, copper, selenium, and iron is associated with growth restriction, pre-eclampsia, early pregnancy loss, and preterm birth [8,9,10,11]. During the highly metabolically active process of pregnancy and placentation, micronutrients act as components of the endogenous antioxidant enzyme systems essential for protecting placental and foetal tissues from oxidative stress and ensuring appropriate vascularisation. The copper/zinc-dependent superoxide dismutase, manganese-dependent superoxide dismutase, iron-dependent catalase, and selenium-dependent glutathione peroxidase are antioxidant enzymes active throughout gestation in the placenta and are depleted in pathological pregnancies [12,13,14]. Essential elements that are necessary for function in larger quantities (grams per day), termed macronutrients, include calcium, potassium, and phosphorous and are integral for foetal bone mineralisation, cell membrane maintenance, and signal transduction [15]. However, in excess, both micro- and macronutrients are related to adverse foetal outcomes, indicative of a narrow window of intake at which these elements are beneficial [16,17]. Transition metal elements such as copper, iron, and zinc can, due to their high reactivity, act as pro-oxidants in excess, a feature they share with non-essential, toxic element pollutants such as arsenic [18]. The placental milieu may be impacted by essential and quasi-essential elements such as molybdenum and bromine and non-essential elements including strontium and rubidium [19,20,21].

Numerous technologies have been established and validated for measuring trace elements (minerals present at very low concentrations in biological tissues) in the placental matrix and maternal and foetal circulations [22,23,24]. Inductively coupled plasma mass spectrometry (ICP-MS), and atomic absorption spectrometry (AAS) [25] are commonly used techniques that rely on the dissolution or digestion of solid samples, typically in dilute acids or alkalis prior to element detection, and allow for high-throughput and multi-element analysis [26]. However, sample preparation is time-consuming, increasing the risk of inaccuracies through contamination, incomplete dissolution, and element content loss, and it does not provide spatial information regarding element abundance and localisation in situ. Synchrotron-based X-ray fluorescence microscopy (XFM) is an attractive alternative to other techniques, as a tool for identification, quantification and spatially distributing trace elements in biological tissue. XFM uses X-rays to excite the atoms of a sample and detects the wavelengths of energy emitted in response; emissions have characteristic energies that allow for element identification [27]. Samples require minimal preparation, are measured at room temperature and pressure in a solid state, and only require low sample volumes. Below certain thresholds, X-rays do not cause tissue degradation [28], which allows for downstream investigations of the same tissue sample.

Since its development in the 1990s, synchrotron-based XFM has been used in diverse fields, including in medical sciences for the examination of human and animal tissue [29]. To date, however, few studies have examined placental tissue specifically via X-ray fluorescence, and those that have, focused on specimen fixation and storage techniques rather than elucidating elemental content in healthy and pathological tissue [30]. Meanwhile, a benchtop variation of this technique that employs X-rays for analysis, known as total reflection X-ray fluorescence spectrometry (TXRF), has been used on microwave-digested or slurry preparation (powdered tissue in suspension) placental tissue, with no spatial data possible [31,32]. 

This feasibility study is the first to examine solid-state cryo-sectioned placental samples to generate elemental maps and determine the abundance of essential and non-essential trace elements in placentae of healthy, postdate, preterm growth-restricted, and stillbirth pregnancies. 

## 2. Materials and Methods

### 2.1. Ethics Statement

Approval from the Human Research Ethics Committee of Hunter New England Health Services, and the University of Newcastle, NSW, Australia, was obtained before the commencement of the project. All research was in accordance with the institutional guidelines. Placental tissue was collected from 19 patients recruited at the John Hunter Hospital (Newcastle, Australia) in the period of 2015–2019. Written, informed consent was obtained from patients by midwives before human placentae were collected from the delivery suite at John Hunter Hospital (Newcastle, NSW, Australia). Confidentiality of participant information was maintained throughout the project.

### 2.2. Sample Collection

Placentae were collected and stratified within specific subgroups, defined as healthy-term controls (*n* = 5, 37–39 weeks), postdates (*n* = 7, >41 completed weeks), preterm FGR (*n* = 3, <32 weeks, birth weight <3rd centile), and term stillbirths (*n* = 4, 37–40 weeks, unexplained). Other maternal characteristics, including age, BMI, and additional complications, were also recorded. Multi-foetal pregnancies and patients <18 years of age were excluded.

### 2.3. Processing of Placental Tissues

Punch biopsies were obtained 4–5 mm beneath the chorionic plate, and tissue samples were immediately frozen under liquid nitrogen and stored at −80 °C. Care was taken to avoid the periphery of the placenta and umbilical cord site; otherwise, the site was randomly selected [33]. Sectioning was performed at −20 °C on a Leica CM1950 cryostat (Hunter Medical Research Institute, Newcastle, Australia), with sections of 60 μm thickness dried on 1 μm thick silicon nitride windows (Australian National Fabrication Facility, University of Queensland). Transfer of the section from the cryostat to the thin film was facilitated by stainless-steel tweezers. Samples were stored at normal air pressure in a cool, dark environment until analysis.

### 2.4. X-ray Fluorescence Microscopy

Element distribution throughout selected 60 μm thick placental sections was mapped at the XFM beamline at the Australian Synchrotron [29]. Samples were continuously scanned in a 20.2 keV X-ray beam focused to a ca. 2 μm full width at half maximum (FWHM) spot using a Kirkpatrick–Baez mirror pair [29]. Fluorescence photons were collected with a 2 μm effective pixel size and 5 ms effective dwell per pixel using a single-element Hitachi Vortex ME3 silicon drift detector (SDD) (Hitachi High-Tech, Tokyo, Japan) mounted at 90 degrees to the incident beam. Spectra analysed using the Dynamic Analysis method as implemented in GeoPIXE (v7.7) [34] produced quantitative 32-bit elemental maps with units either as parts per million (ppm) or weight percent (wt%) depending on the elemental concentration. Note that 10,000 ppm equals 1 wt%. For each sample, 2 × 2 mm portions of uninterrupted tissue were selected for the analysis of distribution and concentration in each placental sample; regions of folded tissue, torn tissue, or visible degradation were excluded. Final regions of interest (ROIs) measured 1.2 × 1.2 mm, with one ROI square per sample, for a total of 19 samples analysed. Data were exported to Excel (v2307) and tabulated.

### 2.5. Statistical Analysis and Representation of Data

GraphPad Prism software (Version 8.4.2) was used to prepare all figures, with individual data points plotted and mean values and standard deviation displayed. Element ranges across all data and median values were also displayed. Only elements detected in all samples were considered for statistical analysis. Data were considered significant with *p* values less than or equal to 0.05 (95% CI). One-way ANOVA was used across all clinical and elemental groups, followed by Tukey’s (patient data) or Bonferroni’s (element abundance) post hoc analyses. Significant changes in element concentrations between healthy term controls and pathological groups are presented. Correlation analyses were performed with a 95% confidence interval and two-tailed *p* values.

## 3. Results

### 3.1. Study Population Characteristics

A total of 19 placental samples across four different experimental groups were selected for analysis (Table 1). Significant differences in the duration of pregnancy were found between term, postdate, and growth-restricted patients, and birth weight was significantly reduced in growth-restricted pregnancies in comparison to other patient groups. Delivery methods, incidence of smoking, asthma, and underlying complications were all recorded.

### 3.2. Elemental Abundance of Placental Samples

The concentration ranges of the 14 elements detected across all placental samples can be found in Supplementary Table S1. Macronutrient elements, including potassium, chlorine, calcium, and phosphorous, exhibited the highest values across all specimens. Of the essential trace elements, the detected median and mean concentrations across all samples, in descending order of abundance, were iron, zinc, bromine, molybdenum, copper, and selenium. Non-essential trace elements detected consistently in placental tissue were rubidium, strontium, and arsenic. Synchrotron-based XFM was sufficient to detect elemental abundance as low as 0.002 ppm for arsenic and as high as 21,818 ppm for sulphur. Manganese, nickel, and chromium were not detected reliably across all samples and therefore were excluded from subsequent analysis.

### 3.3. Comparison of Essential Macronutrients in Healthy and Pathological Placentae

**Figure 1:**
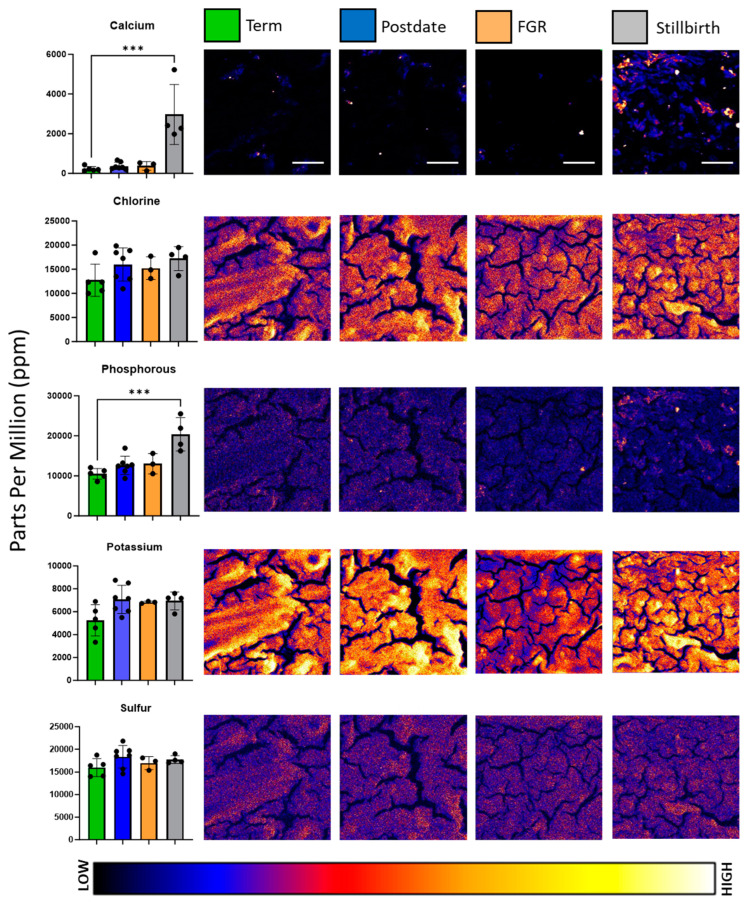
Representative elemental maps of calcium, chlorine, phosphorous, potassium, and sulphur in term, postdate, foetal growth-restricted (FGR), and stillbirth placentae. Colour bar is a visual representation of the intensity of the signal from low (black/blue) to high (yellow/white). Scale bars represent 300 μm and are applicable for all images. Left-hand bar charts indicate the distribution of elemental concentrations per group, with mean and standard deviation indicated. Bar chart colour corresponds to the key at the top of the figure, with green representing healthy term, blue representing postdates, peach representing foetal growth restriction, and grey representing stillbirth. Each elemental map measures 1.2 mm by 1.2 mm. For clarity of image and element abundance, all maps were set to adjust the maximum image value to display as 50% of the maximum; *** signifies *p* < 0.0005.

Maps of macronutrients potassium, phosphorous, chlorine, and calcium distribution and abundance were generated for each sample; representative images are depicted in Figure 1. Variations in signal intensity signified differences in element concentrations across the placental section.

Stratifying macronutrient concentrations by pregnancy complication revealed changes in elemental abundance and reflected the element maps. ANOVA analysis revealed significant differences in calcium (*F* = 15.36, *p* < 0.0001) and phosphorous (*F* = 11.6, *p* = 0.0003) concentrations across complications. Post hoc analysis further identified that calcium and phosphorous were significantly elevated in stillbirth placental tissue in comparison to healthy term. Calcium exhibited some of the greatest variability in the panel of elements analysed, with a mean value of 229.2 ± 119.8 ppm in term samples, in comparison to 2971.75 ± 1512.56 ppm in stillbirth placental tissue. Phosphorous was almost doubled in concentration between healthy term (10,495.4 ± 1343.6 mean ppm) and stillbirth placentae (20,401.0 ± 4160.0 mean ppm).

### 3.4. Comparison of Essential Micronutrients in Healthy and Pathological Placentae

**Figure 2:**
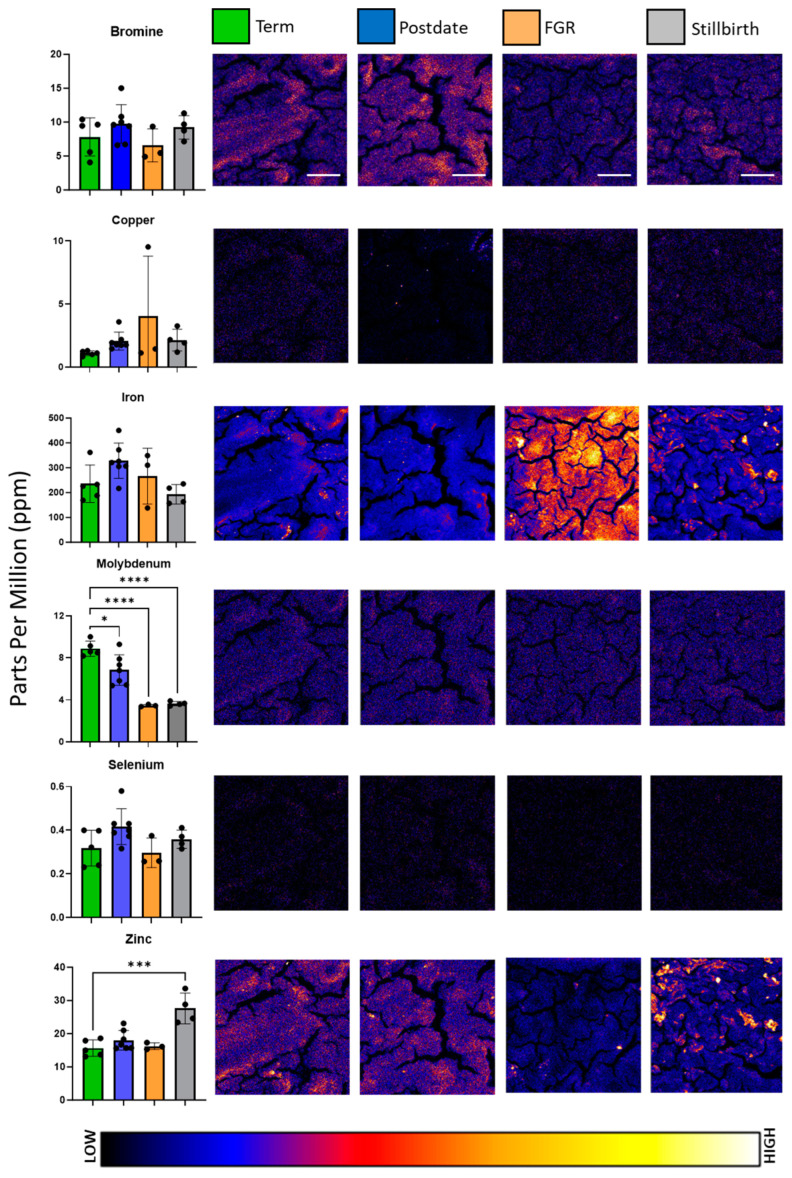
Representative elemental maps of bromine, copper, iron, molybdenum, selenium, and zinc in normal, postdate, foetal growth-restricted (FGR), and stillbirth placentae. Colour bar is a visual representation of the intensity of the signal from low (black/blue) to high (yellow/white). Scale bars represent 300 μm and are applicable for all images. Left-hand bar charts present the distribution of elemental concentrations per group, with mean and standard deviation indicated. Bar chart colour corresponds to the key at the top of the figure, with green representing healthy term, blue representing postdates, peach representing foetal growth restriction, and grey representing stillbirth. Each elemental map measures 1.2 mm by 1.2 mm. For clarity of image and element abundance, all maps were set to adjust the maximum image value to display as 50% of the maximum; * signifies *p* < 0.05, *** signifies *p* < 0.0005; **** signifies *p* < 0.0001.

Maps of the distribution and abundance of micronutrient elements bromine, copper, iron, molybdenum, selenium, and zinc were generated for each sample, and representative images are depicted in Figure 2. In comparison to the macronutrient elemental maps, micronutrients exhibited greater variation in distribution and intensity across samples. Initial ANOVA analysis revealed differences in micronutrient content between experimental groups for molybdenum (*F* = 29.22, *p* < 0.0001) and zinc (*F* = 13.26, *p* = 0.0002). As reflected in the punctate elemental map, zinc concentrations were significantly higher in stillbirth-complicated placentae, at 27.6 ± 4.6 mean ppm, in comparison to healthy term at 15.66 ± 2.47 mean ppm. In contrast, molybdenum was found to be significantly decreased in postdate, foetal growth-restricted, and stillbirth samples, at 6.83 ± 1.45, 3.46 ± 0.11, and 3.64 ± 0.2, respectively, in comparison to healthy term, at 8.87 ± 0.72 mean ppm in the subsequent post hoc analysis. Molybdenum was the only element evaluated that decreased in pathologically complicated placentae, despite its low abundance and uniform distribution. No significant differences in concentrations of selenium, copper, bromine, or iron were found between experimental groups, overall. However, punctae of increased zinc and iron accumulation were noted in stillbirth samples.

### 3.5. Comparison of Non-Essential Elements in Healthy and Pathological Placentae

**Figure 3:**
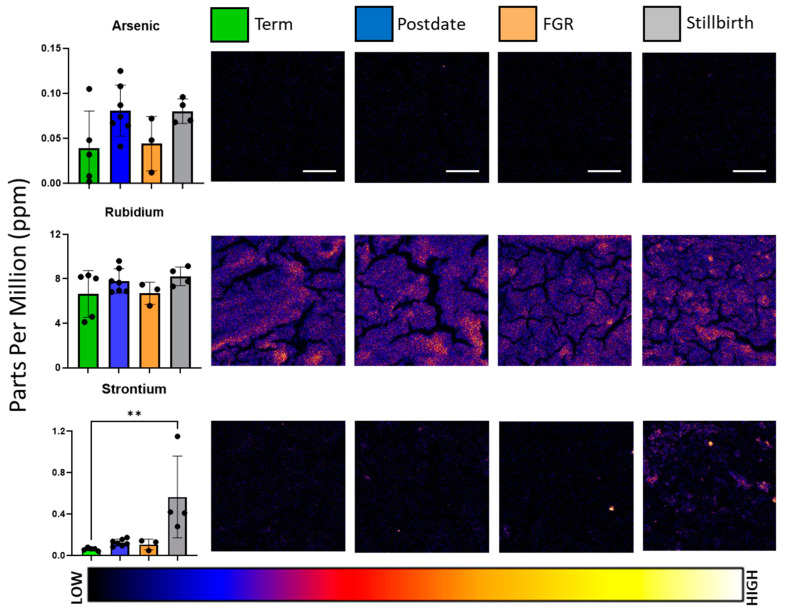
Representative elemental maps of arsenic, rubidium, and strontium, in normal, postdate, foetal growth-restricted (FGR), and stillbirth placentae. Colour bar is a visual representation of the intensity of the signal from low (black/blue) to high (yellow/white). Scale bars represent 300 μm and are applicable for all images. Left-hand bar charts present the distribution of elemental concentrations per group, with mean and standard deviation indicated. Bar chart colour corresponds to the key at the top of the figure, with green representing healthy term, blue representing postdates, peach representing foetal growth restriction, and grey representing stillbirth. Each elemental map measures 1.2 mm by 1.2 mm. For clarity of image and element abundance, all maps were set to adjust the maximum image value to display as 50% of the maximum; ** signifies *p* < 0.005.

The pollutant element arsenic and non-essential elements strontium and rubidium were also present in placental tissue sections, as depicted in Figure 3, but notably, only rubidium was observed in concentrations higher than 1 ppm. ANOVA analysis revealed that strontium was significantly altered between experimental groups (*F* = 7.225, *p* = 0.0032), with healthy-term samples at 0.061 ± 0.014 mean ppm vs. stillbirth tissue at 0.565 ± 0.395 mean ppm. Arsenic accumulation was observed in postdate and stillbirth placentae, whose average concentrations reached 0.081 ± 0.03 ppm and 0.08 ± 0.014 ppm, respectively, higher than term samples at 0.039 ± 0.04 ppm, but the difference was not significant.

### 3.6. Correlation Analysis of Essential and Non-Essential Elements in Placental Tissue

**Figure 4:**
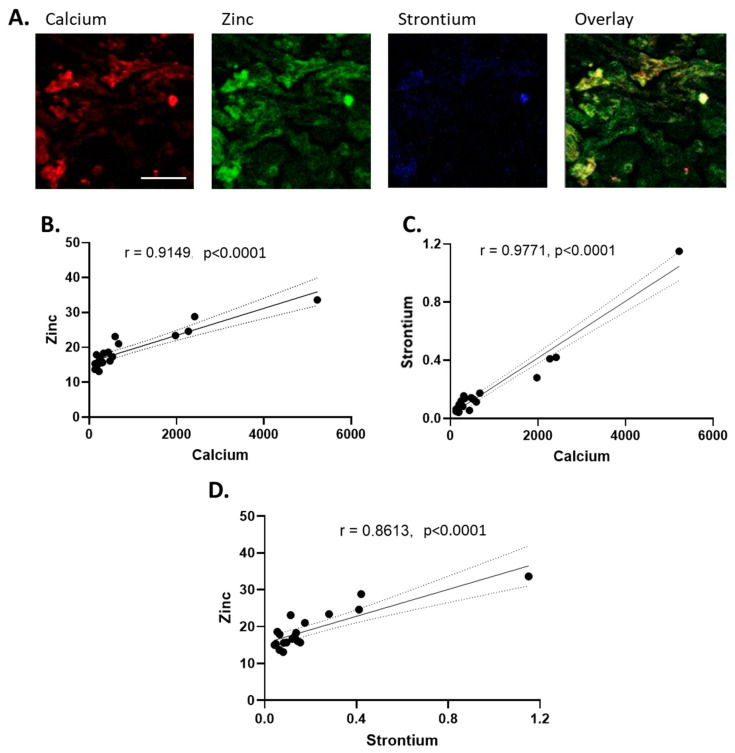
(**A**) Representative false-colour elemental maps of calcium (red), zinc (green), and strontium (blue), and overlay in a stillbirth placenta. Scale bar represents 200 μm and is applicable for all images. Each elemental map measures 0.7 mm by 0.7 mm. For clarity of image and element abundance, all maps were set to adjust the maximum image value to display as 50% of the maximum. The Pearson correlation test was performed with a 95% confidence interval for (**B**) zinc vs. calcium, (**C**) strontium vs. calcium, and (**D**) strontium vs. zinc. R values and *p* values are displayed.

A relationship between calcium and strontium, as well as calcium and zinc, was observed in individual maps of stillbirth tissue, and the results are presented below via a three-element false-colour analysis of a representative stillbirth sample (Figure 4A). The correlation analysis of concentration values for all placental groups revealed positive relationships between zinc and calcium (Figure 4B), strontium and calcium (Figure 4C), and zinc and strontium (Figure 4D), with r values of 0.9149 (*p* < 0.0001), 0.9771 (*p* < 0.0001), and 0.8613 (*p* < 0.0001), respectively. Analysis was subsequently performed on all experimental groups, excluding stillbirth, to evaluate whether the correlation of the observed elements was the result of stillbirth samples. The r values of the remaining 15 samples were 0.6712 between strontium and calcium (*p* = 0.0062) and 0.7625 between calcium and zinc (*p* = 0.0009). Strontium and zinc were no longer significantly correlated, with an r value of 0.4185 (*p* = 0.1205).

## 4. Discussion

This feasibility study evaluated the concentration and distribution of elements via X-ray fluorescence microscopy in placental tissue from healthy, growth-restricted, postdate, and stillbirth-complicated pregnancies. Synchrotron-based XFM allowed for multi-element analysis of tissue with sensitivity as low as parts per billion, from a minimal sample volume, and generated both quantitative and co-localisation data of elements in situ. 

A range of analytical techniques have been used to evaluate placental element content in the past few decades and generated foundational data for epidemiological, human biomonitoring, and placental physiology research [23]. In comparison to other analytical techniques such as ICP-MS, FAAS, and INAA, XFM provided similar patterns of relative abundance (Table 2), supporting our methodology in utilising synchrotron-based XFM for the analysis of placental tissue.

Furthermore, our synthesis of the literature revealed wide inter-variation in element concentrations, even within healthy-term populations. The international scope of the literature, spanning cohorts from Poland, Jamaica, America, Greece, and Serbia, amongst others, is an integral and unavoidable contributor to variability, as differences in element content in soil and foodstuffs, as well as seasonal differences in diet, have been shown to affect nutrient intake and element exposure [35,36]. However, inherent differences in analytical techniques, including detection limits, element/wavelength identification between methods, and sample drift, also need to be taken into consideration when variance is found, as should sample preparation (aerosolisation vs. microwave digestion vs. solid-state sampling) and data reporting (dry vs. wet weight). In addition, intra-placental differences as a result of the heterogeneity of placental structures, which are undetectable by the majority of these techniques, could skew element data between studies [33]. The capability of the XFM beamline to generate high-resolution element distribution maps, in conjunction with the minimal processing of placental tissue in this study, allowed for the observation of element trends and co-localisation, which can be further combined with molecular analyses to elucidate cellular structures. 

**Table 2:** Summary of element ranges in healthy control placental tissue, from the current study in comparison with the existing literature.

Element	Synchrotron Values (Min–Max ppm)	Combined Research Values (Min–Max ppm)	Methods of Detection	References
Arsenic	0.002–0.105	0.00006–0.17	ICP-MS; ICP-OES; INAA	[20,22,23,37]
Bromine	4.08–10.4	2.8–52.7	INAA	[21,38]
Calcium	132–435	67.3–54,480	ICP-MS; ICP-OES; FAAS	[20,39,40]
Copper	0.83–1.3	0.05–15.391	ICP-MS; ICP-OES; FAAS; ICP-AES; AAS	[20,22,39,40,41]
Iron	169–362	22.2–1648	FAAS; ICP-AES; AAS; ICP-MS	[20,39,40,41]
Molybdenum	8.17–10	0.0061–0.170	ICP-AES; ICP-OES; ICP-MS	[8,22,40]
Phosphorous	8559–12,069	3540–14,300	ICP-OES	[20]
Potassium	3340–6892	115–10,093.3	ICP-OES; FAAS	[20,39]
Rubidium	4.1–8.3	0.42–19.6	ICP-MS; ICP-OES; INAA	[20,21,22,38]
Selenium	0.23–0.4	0.0834–2.67	INAA; ICP-MS;	[21,22,23,37]
Strontium	0.043–0.08	0.0406–16.990	ICP-AES; ICP-MS	[22,40]
Zinc	13.1–18.6	3.3–109.288	FAAS; ICP-AES; AAS; ICP-MS	[22,23,39,40,41]

Data presented as ppm, converted from original study units. Abbreviations: ICP-MS, inductively coupled plasma mass spectrometry; ICP-OES, inductively coupled plasma optical emission spectroscopy; INAA, instrumental neutron activation analysis; (F)AAS, (flame) atomic absorption spectrometry; ICP-AES, inductively coupled plasma atomic emission spectrometry.

Macronutrient elements such as chlorine, potassium, and sulphur exhibited gradual changes in signal intensity, which we speculate parallel the placental villous structure. Calcium abundance was low across the observed placental tissues except for high-intensity puncta, the majority of which were observed in the stillbirth sections. Concurrently, of the 19 samples analysed, significant differences in element content were noted primarily in stillbirth-complicated placentae, which spatially and quantitatively were associated with increased calcium-based nodules in the tissue. While the literature on stillbirth tissue is rare, the calcification of the placenta, which involves the deposition of calcium–phosphate minerals, is associated with poor maternal and perinatal outcomes, notably in the development of pre-eclampsia and IUGR [16]. Previous use of X-ray fluorescence-based analytics revealed that strontium co-localises with calcium in calcium-based stones and validated the use of strontium as a marker for studying calcium lithogenesis [42]. Our study was the first to analyse the co-localisation of strontium and calcium in placental tissue and may shed light on the calcification processes of the placenta. Increased levels of strontium in maternal serum were also recently associated with vascular complications such as pre-eclampsia during pregnancy [43]; however, no significant differences were found in the concentration of strontium between this study’s healthy and growth-restricted experimental groups. The co-localisation of zinc with calcium and strontium was also visualised in situ. While a significant correlation between zinc and calcium existed across all samples, zinc and strontium were no longer significantly correlated with the removal of stillbirth-complicated pregnancies from analysis, suggesting that different physiological processes lead to this co-localisation and calcium build-up in different pathologies. Considering the nature of stillbirth tissue, however, it is difficult to ascertain whether this change in element concentrations was a precursor to the pathology or a result of it.

In comparison to the macronutrient elemental maps, micronutrients exhibited greater variation in distribution and intensity across samples. Micronutrients such as selenium, manganese, copper, zinc, and iron, which are vital co-factors for the function of several antioxidant enzyme systems, are highly represented in the literature on placental and gestational health. However, the relationship between essential trace element content and the plethora of gestational complications studied has not been fully elucidated, and the literature is at times contradictory. Both high and low selenium concentrations in placental tissue and maternal circulation have been associated with foetal growth restriction, hypertensive disorders of pregnancy, and preterm birth [44,45,46]. Copper and iron, in deficiency and excess, lead to oxidative stress and have been linked to spontaneous miscarriage, hypertension, gestational diabetes, and preterm birth [47]. Decreased circulating and placental content of zinc has been linked to reduced birth weight, pre-eclampsia, and IUGR [23,44,48], while excessive intake of one element, via diet or cigarette consumption, can also lead to the reduced absorption of other essential micronutrients or impede placental transfer via accumulation [39,49,50]. Synchrotron analysis could explore the potential for differences in trace element levels to correlate with the protein expression of key antioxidant enzymes and examine their distribution across placental structures and organelles. Identifying the punctae of pollutant element aggregation, such as the established carcinogen arsenic [51], could also provide information regarding sites of oxidative stress in placental tissue. 

Molybdenum was unique in this study as a consistently and significantly decreased element across all pathological groups. Of the few studies that performed multi-element analysis including molybdenum, the recorded concentrations were significantly lower than the values reported via XFM but remained heterogenous. Molybdenum forms the co-factor of the enzymes aldehyde, xanthine, and sulphite oxidase (AOX, XO, and SO, respectively), and mitochondrial amidoxime reducing components 1 and 2 (MARC1, MARC2), which are key players in the metabolism of aldehydes, purines, and sulphur-containing amino acids [19]. Molybdoenzyme activity has been associated with increased oxidative stress; placental ageing; and the development of chronic conditions, including obesity and cancer [52,53]. More recently, in contrast, molybdenum deficiency has been linked to an increased incidence of metabolic disorders and neural tube defects during gestation when examining maternal blood and amniotic fluid [17,19]. This may be indicative of a narrow beneficial range for molybdenum in placental function; this research is one of the first to examine the role of molybdenum in pregnancy health and specifically molybdenum content in multiple different pathologies via synchrotron-based XFM.

This research provides proof of concept for the methodology of cryo-sectioning and freeze-drying placental samples for analysis by synchrotron beam. Previous research examining placental tissue via X-ray fluorescence discovered that commonly used sample preservation methods, such as the use of formaldehyde and glutaraldehyde for fixation, could induce metal redistribution, leeching, and exogenous absorption of elements [30]. Overall, the ease of sample preparation, non-destructive technique, reduced risk of contamination, and ability to generate multiple forms of data from each sample highlight synchrotron-based XFM as an exciting and promising alternative to other methods.

### Limitations of the Study

This study has several limitations. Firstly, while synchrotron-based X-ray fluorescence microscopy is a valuable and powerful tool for multi-element analysis, it is not yet suitable for high-throughput analysis due to the scan time per sample and competitive beamtime hours, which affected our sample size. Moreover, certain elements such as manganese were below the limit of detection via this method in comparison to other analytical instruments such as ICP-MS in placental tissue [22]. This may be related to the nature of specific elements when interrogated via a synchrotron beam; each element has an inherent fluorescence yield, and manganese has previously been reported as inconsistently detected in synchrotron-based XFM studies [54]. Therefore, careful consideration is required to ensure that the XFM beamline is utilised appropriately. 

Aside from technical considerations, we acknowledge that the data generated in this study are the result of a single beamtime experiment and are therefore preliminary. In addition, constraints regarding the availability of tissue samples, notably from complicated pregnancies, contribute to the small study size and may introduce selection bias and sample bias. It is likely that an increase in the sample number would elucidate further significant differences in elemental content. Furthermore, tissue samples were collected from a single site for each placenta, which may not accurately reflect the complex, spatially dependent interplay of element transfer and accumulation that is observed within this 500 g organ. Future research could include sampling multiple sites to evaluate intra-placental element content via XFM. 

There are also limited data in the literature for the element content of stillbirth and postdate placentae with which to compare our results specifically. Stillbirth placental tissue is understandably difficult to metabolically assess, as numerous secondary changes occur following the cessation of the fetoplacental circulation as a result of intrauterine foetal death, which can confound results [33]. Lastly, this study was not able to use histological analysis in tandem with XFM to provide information on the underlying structures that influenced element content or transfer and storage, therefore not utilising these data to the fullest capacity. We speculate that a number of elements in our analysis parallel the placental villous structure, which subsequent studies can investigate. 

With an increased sample size, the addition of histological information for each sample, and the validation of the quantitative data via an alternative technique such as ICP-MS, XFM can present fresh insights into this essential organ.

## 5. Conclusions

In this study, XRF imaging was used for the first time to compare the concentrations of essential elements quantitatively and qualitatively in cryo-sectioned placental tissues. Further, this is the first analysis using this technique for the assessment of three separate pathological pregnancy groups, including stillbirth and postdate tissues. Elemental maps revealed both uniform and patterned distributions of essential micronutrients. The transition metal and essential enzyme co-factor molybdenum was found to be significantly decreased in all gestational pathologies. This study can provide foundational context for continuing analyses of placental tissue. 

## Figures and Tables

**Table 1:** Demographic and clinical characteristics of placentae.

		Term, a *n* = 5	Postdates, b *n* = 7	FGR, c *n* = 3	Stillbirth, d *n* = 4	ANOVA *F*, *p* Value	Pairwise Comparison	
Weeks’ Gestation		37.94 ± 0.74	41.34 ± 0.2	32.23 ± 2.28	39.1 ± 1.23	51.56, <0.0001	a–b	0.0004
							a–c	<0.0001
							b–c	<0.0001
							b–d	0.0213
							c–d	<0.0001
Birthweight (g)		3212 ± 518.91	3690 ± 556.66	1350.0 ± 296.14	3387.5 ± 533.82	15.13, <0.0001	a–c	0.0009
							b–c	<0.0001
							c–d	0.0006
Infant Sex	Female	4	5	2	2			
	Male	1	2	1	2			
Delivery Method	Vaginal	3	5	0	4			
	Emergency Caesarean	1	2	3	0			
	Elective Caesarean	1	0	0	0			
Maternal BMI		21.014 ± 4.25	27.46 ± 6.64	30.01 ± 2.78	23.66 ± 4.31			
Maternal Age		29.24 ± 4.3	29.49 ± 5.06	26.87 ± 3.24	30.28 ± 6.08			
Smoked During Pregnancy	Yes	0	1	2	0			
	No	5	6	1	4			
Asthma		0	3	2	1			
Depression (Treated)		0	1	0	0			
Pre-eclampsia		0	0	2	0			
Anaemia		1	1	0	0			
Steroid Treatment		0	0	3	0			
Fatty Liver Disease		0	0	1	0			
PCOS		0	1	1	0			

Data are presented as mean ± standard deviation, or percentage (%). Abbreviations: FGR, foetal growth restriction, <32 weeks, birth weight <3rd centile; PCOS, polycystic ovarian syndrome.

## Data Availability

Dataset is available upon request from the authors.

## Supplementary Materials

The following supporting information can be downloaded at: https://www.mdpi.com/article/10.3390/nu16152549/s1: Table S1, Elemental abundance ranges for selected elements in all samples, ordered from the highest to the lowest concentration (mean).

## References

[1] Burton G.J., Fowden A.L., Thornburg K.L. (2016). Placental Origins of Chronic Disease. Physiol. Rev..

[2] Lean S.C., Heazell A.E.P., Dilworth M.R., Mills T.A., Jones R.L. (2017). Placental Dysfunction Underlies Increased Risk of Fetal Growth Restriction and Stillbirth in Advanced Maternal Age Women. Sci. Rep..

[3] Gagnon R. (2003). Placental insufficiency and its consequences. Eur. J. Obstet. Gynecol. Reprod. Biol..

[4] Bastek J.A., Brown A.G., Anton L., Srinivas S.K., D’Addio A., Elovitz M.A. (2011). Biomarkers of inflammation and placental dysfunction are associated with subsequent preterm birth. J. Matern.-Fetal Neonatal Med..

[5] Barker D.J.P. (1995). Intrauterine programming of adult disease. Mol. Med. Today.

[6] Neiger R. (2017). Long-Term Effects of Pregnancy Complications on Maternal Health: A Review. J. Clin. Med..

[7] Mousa A., Naqash A., Lim S. (2019). Macronutrient and Micronutrient Intake during Pregnancy: An Overview of Recent Evidence. Nutrients.

[8] Gómez-Roig M.D., Mazarico E., Cuadras D., Muniesa M., Pascal R., Ferrer P., Cantallops M., Arraez M., Gratacós E., Falcon M. (2021). Placental chemical elements concentration in small fetuses and its relationship with Doppler markers of placental function. Placenta.

[9] Kurlak L.O., Scaife P.J., Briggs L.V., Broughton Pipkin F., Gardner D.S., Mistry H.D. (2023). Alterations in Antioxidant Micronutrient Concentrations in Placental Tissue, Maternal Blood and Urine and the Fetal Circulation in Pre-eclampsia. Int. J. Mol. Sci..

[10] Mistry H.D., Williams P.J. (2011). The Importance of Antioxidant Micronutrients in Pregnancy. Oxidative Med. Cell. Longev..

[11] McKeating D.R., Fisher J.J., Perkins A.V. (2019). Elemental Metabolomics and Pregnancy Outcomes. Nutrients.

[12] Wang Y., Walsh S.W. (1996). Antioxidant Activities and mRNA Expression of Superoxide Dismutase, Catalase, and Glutathione Peroxidase in Normal and Preeclamptic Placentas. J. Soc. Gynecol. Investig..

[13] Ghneim H.K., Alshebly M.M. (2015). Biochemical Markers of Oxidative Stress in Saudi Women with Recurrent Miscarriage. J. Korean Med Sci..

[14] Richard K., Holland O., Landers K., Vanderlelie J.J., Hofstee P., Cuffe J.S.M., Perkins A.V. (2017). Review: Effects of maternal micronutrient supplementation on placental function. Placenta.

[15] Stenhouse C., Suva L.J., Gaddy D., Wu G., Bazer F.W., Wu G. (2022). Phosphate, Calcium, and Vitamin D: Key Regulators of Fetal and Placental Development in Mammals. Recent Advances in Animal Nutrition and Metabolism.

[16] Chen K.H., Chen L.R., Lee Y.H. (2011). Exploring the relationship between preterm placental calcification and adverse maternal and fetal outcome. Ultrasound Obstet. Gynecol..

[17] Huang W., Fu J., Yuan Z., Gu H. (2023). Impact of prenatal exposure to metallic elements on neural tube defects: Insights from human investigations. Ecotoxicol. Environ. Saf..

[18] Stojsavljević A., Perović M., Nešić A., Miković Ž., Manojlović D. (2022). Levels of non-essential trace metals and their impact on placental health: A review. Environ. Sci. Pollut. Res..

[19] Foteva V., Fisher J.J., Qiao Y., Smith R. (2023). Does the Micronutrient Molybdenum Have a Role in Gestational Complications and Placental Health?. Nutrients.

[20] Rduch T., Tsolaki E., El Baz Y., Leschka S., Born D., Kinkel J., Anthis A.H.C., Fischer T., Jochum W., Hornung R. (2022). The Role of Inorganics in Preeclampsia Assessed by Multiscale Multimodal Characterization of Placentae. Front. Med..

[21] Alexiou D., Grimanis A.P., Grimani M., Papaevangelou G., Koumantakis E., Papadatos C. (1977). Trace Elements (Zinc, Cobalt, Selenium, Rubidium, Bromine, Gold) in Human Placenta and Newborn Liver at Birth. Pediatr. Res..

[22] Stojsavljević A., Rovčanin M., Rovčanin B., Miković Ž., Jeremić A., Perović M., Manojlović D. (2021). Human biomonitoring of essential, nonessential, rare earth, and noble elements in placental tissues. Chemosphere.

[23] Mikelson C.K., Troisi J., LaLonde A., Symes S.J.K., Thurston S.W., DiRe L.M., David Adair C., Miller R.K., Richards S.M. (2019). Placental concentrations of essential, toxic, and understudied metals and relationships with birth outcomes in Chattanooga, TN. Environ. Res.

[24] McKeating D.R., Fisher J.J., MacDonald T., Walker S., Tong S., Bennett W.W., Kaitu’u-Lino T.J., Perkins A.V. (2021). Circulating trace elements for the prediction of preeclampsia and small for gestational age babies. Metabolomics.

[25] Al-Saleh I., Shinwari N., Mashhour A., Mohamed G.E.D., Rabah A. (2011). Heavy metals (lead, cadmium and mercury) in maternal, cord blood and placenta of healthy women. Int. J. Hyg. Environ. Health.

[26] Wilschefski S.C., Baxter M.R. (2019). Inductively Coupled Plasma Mass Spectrometry: Introduction to Analytical Aspects. Clin. Biochem. Rev..

[27] Verma H. (2007). X-ray fluorescence (XRF) and particle-induced X-ray emission (PIXE). Atomic and Nuclear Analytical Methods: XRF, Mössbauer, XPS, NAA and B63Ion-Beam Spectroscopic Techniques.

[28] Jones M.W.M., Hare D.J., James S.A., de Jonge M.D., McColl G. (2017). Radiation Dose Limits for Bioanalytical X-ray Fluorescence Microscopy. Anal. Chem..

[29] Howard D.L., de Jonge M.D., Afshar N., Ryan C.G., Kirkham R., Reinhardt J., Kewish C.M., McKinlay J., Walsh A., Divitcos J. (2020). The XFM beamline at the Australian Synchrotron. J. Synchrotron Radiat..

[30] Punshon T., Chen S., Finney L., Howard L., Jackson B.P., Karagas M.R., Ornvold K. (2015). High-resolution elemental mapping of human placental chorionic villi using synchrotron X-ray fluorescence spectroscopy. Anal. Bioanal. Chem..

[31] Hauser S., Andres S., Leopold K. (2022). Determination of trace elements in placenta by total reflection X-ray fluorescence spectrometry: Effects of sampling and sample preparation. Anal. Bioanal. Chem..

[32] Marguí E., Ricketts P., Fletcher H., Karydas A.G., Migliori A., Leani J.J., Hidalgo M., Queralt I., Voutchkov M. (2017). Total reflection X-ray fluorescence as a fast multielemental technique for human placenta sample analysis. Spectrochim. Acta Part B At. Spectrosc..

[33] Burton G.J., Sebire N.J., Myatt L., Tannetta D., Wang Y.L., Sadovsky Y., Staff A.C., Redman C.W. (2014). Optimising sample collection for placental research. Placenta.

[34] Ryan C.G., Etschmann B.E., Vogt S., Maser J., Harland C.L., van Achterbergh E., Legnini D. (2005). Nuclear microprobe—Synchrotron synergy: Towards integrated quantitative real-time elemental imaging using PIXE and SXRF. Nucl. Instrum. Methods Phys. Res. Sect. B Beam Interact. Mater. At..

[35] Silver W.L., Perez T., Mayer A., Jones A.R. (2021). The role of soil in the contribution of food and feed. Philos. Trans. R. Soc. Lond. B Biol. Sci..

[36] Moreno-Jiménez E., Maestre F.T., Flagmeier M., Guirado E., Berdugo M., Bastida F., Dacal M., Díaz-Martínez P., Ochoa-Hueso R., Plaza C. (2023). Soils in warmer and less developed countries have less micronutrients globally. Glob. Chang. Biol..

[37] Llanos M.N., Ronco A.M. (2009). Fetal growth restriction is related to placental levels of cadmium, lead and arsenic but not with antioxidant activities. Reprod. Toxicol..

[38] Grant C., Lalor G., Fletcher H., Potter T., Vutchkov M., Reid M. (2010). Elements in human placentae in Jamaica. West Indian Med. J..

[39] Mazurek D., Łoźna K., Bronkowska M. (2020). The concentration of selected elements in the placenta according to selected sociodemographic factors and their effect on birth mass and birth length of newborns. J. Trace Elem. Med. Biol..

[40] Kot K., Kosik-Bogacka D., Łanocha-Arendarczyk N., Malinowski W., Szymański S., Mularczyk M., Tomska N., Rotter I. (2019). Interactions between 14 Elements in the Human Placenta, Fetal Membrane and Umbilical Cord. Int. J. Environ. Res. Public Health.

[41] Mbofung C.M.F., Subbarau V.V. (1990). Trace element (Zinc, copper, iron and magnesium) concentrations in human placenta and their relationship to birth weight of babies. Nutr. Res..

[42] Blaschko S.D., Chi T., Miller J., Flechner L., Fakra S., Kapahi P., Kahn A., Stoller M.L. (2013). Strontium substitution for calcium in lithogenesis. J. Urol..

[43] Barneo-Caragol C., Martínez-Morillo E., Rodríguez-González S., Lequerica-Fernández P., Vega-Naredo I., Álvarez F.V. (2019). Increased serum strontium levels and altered oxidative stress status in early-onset preeclampsia. Free Radic. Biol. Med..

[44] Zadrożna M., Gawlik M., Nowak B., Marcinek A., Mrowiec H., Walas S., Wietecha-Posłuszny R., Zagrodzki P. (2009). Antioxidants activities and concentration of selenium, zinc and copper in preterm and IUGR human placentas. J. Trace Elem. Med. Biol..

[45] Osada H., Watanabe Y., Nishimura Y., Yukawa M., Seki K., Sekiya S. (2002). Profile of trace element concentrations in the feto-placental unit in relation to fetal growth. Acta Obstet. Gynecol. Scand..

[46] Duntas L.H. (2020). Selenium and at-risk pregnancy: Challenges and controversies. Thyroid Res..

[47] Grzeszczak K., Kwiatkowski S., Kosik-Bogacka D. (2020). The Role of Fe, Zn, and Cu in Pregnancy. Biomolecules.

[48] Wilson R.L., Grieger J.A., Bianco-Miotto T., Roberts C.T. (2016). Association between Maternal Zinc Status, Dietary Zinc Intake and Pregnancy Complications: A Systematic Review. Nutrients.

[49] Gernand A.D. (2019). The upper level: Examining the risk of excess micronutrient intake in pregnancy from antenatal supplements. Ann. N. Y. Acad. Sci..

[50] Ronco A.M., Arguello G., Muñoz L., Gras N., Llanos M. (2005). Metals content in placentas from moderate cigarette consumers: Correlation with newborn birth weight. Biometals.

[51] Martinez V.D., Lam W.L. (2021). Health Effects Associated With Pre- and Perinatal Exposure to Arsenic. Front. Genet..

[52] Qiao Y., Maiti K., Sultana Z., Fu L., Smith R. (2020). Inhibition of vertebrate aldehyde oxidase as a therapeutic treatment for cancer, obesity, aging and amyotrophic lateral sclerosis. Eur. J. Med. Chem..

[53] Maiti K., Sultana Z., Aitken R.J., Morris J., Park F., Andrew B., Riley S.C., Smith R. (2017). Evidence that fetal death is associated with placental aging. Am. J. Obstet. Gynecol..

[54] Ceko M.J., Hummitzsch K., Hatzirodos N., Rodgers R.J., Harris H.H. (2015). Quantitative elemental analysis of bovine ovarian follicles using X-ray fluorescence imaging. Metallomics.

