# Supplementary material for: Placental Element Content Assessed via Synchrotron-Based X-ray Fluorescence Microscopy Identifies Low Molybdenum Concentrations in Foetal Growth Restriction, Postdate Delivery and Stillbirth
Source: Nutrients. 2024 Aug 3;16(15):2549. doi: 10.3390/nu16152549 (PMC11314477; doi:10.3390/nu16152549)
Supplement: Supplementary file 1 [file nutrients-16-02549-s001.zip › nutrients-3100519-supplementary.pdf]

Supplementary Table S1. Elemental abundance ranges for selected elements in all samples, ordered highest to lowest concentration (mean)

Element	Min-Max	Median	Mean ± SD
Sulfur	14004-21818	17414	17394.11 ± 2000.45
Chlorine	9984-19838	14869	15263.63 ± 3215.73
Phosphorous	8559-25520	12458	13762.63 ± 4255.40
Potassium	3340-8756	6833	6526.05 ± 1248.71
Calcium	132-5224	329	883.84 ± 1243.34
Iron	138-450	235	265.58 ± 84.50
Zinc	13.1-33.6	17.3	19.13 ± 5.26
Bromine	4.08-15	9.4	8.63 ± 2.58
Rubidium	4.11-9.61	7.65	7.41 ± 1.4
Molybdenum	3.36-10	5.8	6.16 ± 2.32
Copper	0.83-9.54	1.73	2.13 ± 1.88
Selenium	0.231-0.58	0.373	0.36 ± 0.081
Strontium	0.043-1.15	0.12	0.198 ± 0.249
Arsenic	0.002-0.125	0.068	0.064 ± 0.034

All values presented as parts per million (10,000 ppm equals 1 wt%). Range is minimum to maximum values detected per element. SD is standard deviation.
